# Cyclic Fatigue Failure of Perforated 3D-Printed Polylactide (PLA) Specimens by Inserted Pin Loading

**DOI:** 10.3390/ma17225394

**Published:** 2024-11-05

**Authors:** J. S. Hertel, Y. W. Kwon, D. Sachau

**Affiliations:** 1 Department of Mechanical & Aerospace Engineering, Naval Postgraduate School, Monterey, CA 93943, USA; 2 Fakultät Maschinenbau, Helmut-Schmidt-Universität/Universität der Bundeswehr Hamburg, 22043 Hamburg, Germany

**Keywords:** pin loading, cyclic fatigue, 3D printing, polylactide (PLA)

## Abstract

The failure of 3D-printed Polylactide (PLA) specimens with circular holes was studied under tensile and cyclic loading, respectively, by an inserted pin. Experiments were conducted for the perforated PLA specimens with various print angles from 0° to 90°, as well as [0°/90°]s and [0°/±45°/90°]s. The hole locations varied along the specimens. The PLA specimens showed two different failure modes: one through the print lines and the other between the print lines. Different print angles resulted in different tensile failure stresses under pin loading. The cyclic tests of different print angles showed very similar S-N data as the applied stresses were normalized to their tensile failure stresses if the failure mode was through the print lines. On the other hand, cyclic failure between print lines showed distinctly separated S-N data, even with the normalized applied stresses. The tensile failure stresses, failure locations, and orientations were successfully predicted using the failure criterion that is based on both stress and stress gradient conditions. A proposed mathematical interpolation equation provided good estimations of the tensile failure stresses and S-N curves of specimens with different print angles once the failure stresses were known for the 0° to 90° specimens.

## 1. Introduction

Most structural members have notches necessary for their functions. For example, mechanical fasteners require holes in structural members. Notches reduce the load-carrying capability of the structural members because of stress concentration. In addition, inserted pin loading through holes like bolted or rivet joints diminishes the maximum applied load much more than remote loading away from the holes. As a result, extensive research has been conducted to determine the effect of any notches on failure, experimentally or analytically. Prior research has focused on understanding the failure mechanisms/modes or predicting failure.

Traditional failure criteria were developed to predict the failure under multiaxial loading using the uniaxially tested strength value. Various failure criteria have been proposed, depending on the material behavior, such as being brittle, ductile, isotropic, anisotropic, etc. [1,2,3,4]. However, those traditional failure criteria could not predict the failure of specimens with holes. In other words, even though the peak stress with stress concentration at the edge of a hole reaches the failure strength of the material obtained from the standard tensile coupon test, the specimen does not fail. If the notch is not a crack, fracture mechanics [5,6] cannot be applied to the specimen either.

As a result, different failure criteria have been proposed for specimens with holes [7,8,9,10,11,12,13,14,15,16,17,18]. The majority of the failure criteria used the stresses at some distance away from the edge of the hole, where failure is expected. Either the stresses at the selected point or the average stresses from the selected point to the edge of the hole were used in those failure criteria. Much research has focused on finding the location of the so-called critical distance [9,10,12,13]. Those failure criteria implicitly require the failure location and path because the critical location is determined along the failure path [8,11]. However, the failure path is not straightforward to predict for inserted pin loading because the path is not necessarily perpendicular to the uniaxial loading.

A recently proposed failure criterion considers both stress and stress gradient conditions to determine failure [14,15,16,17,18]. Both conditions must be satisfied together for failure to occur. This criterion does not require any critical distance. Furthermore, the failure path is determined from the stress gradient condition. The recent failure criterion predicted the failure of various isotropic or anisotropic materials with different sizes and shapes of notches [14,15,16,17,18].

The recent failure criterion also demonstrated that a specimen with a very tiny hole compared to the specimen width does not initiate failure at the edge of the hole under uniaxial loading [17,18]. Even though the induced stress is the largest at the edge of the hole because of stress concentration, the stress gradient condition is not satisfied there. Instead, both stress and stress gradient conditions are satisfied at a distance away from the tiny hole. This was validated recently by comparing the theoretical prediction to experimental test results [17,18]. This also explains why the presence of a tiny hole does not affect the specimen’s failure load. Furthermore, the failure criterion successfully predicted the tensile failure load and failure path of the polymethyl methacrylate (PMMA) specimens subjected to inserted pin loading. The PMMA material is isotropic.

Additive manufacturing or 3D-printing is a process that involves building multiple individual layers on top of each other to create a part. It can be used with various materials and manufacturing techniques [19]. The components produced through this method have a wide range of applications, including aviation, aerospace, automotive industries, medicine, construction, and the food industry [20,21]. Fused Deposition Molding (FDM), also known as Fused Filament Fabrication (FFF), is one of the most commonly used methods for 3D printers. This process involves heating thermoplastics and applying them to the print bed through the printer nozzle. The thermoplastic is heated to a semi-liquid state, allowing for the bonding of individual layers, which then cool down without further deformation. Some 3D-printed materials like Polylactide (PLA) can manifest orthotropic strength, depending on the printing condition. The strength along the print direction is much higher than that normal to the print direction. That is, the strength between print lines is much weaker than the strength of the print lines. In other words, those specimens behave like unidirectional fibrous composites. Thus, those specimens are useful for investigating the failure of orthotropic materials with two different failure modes. The PLA was selected in this study.

The prior discussion on the various static failure criteria is still applicable to the 3D-printed specimens. In particular, the failure criteria developed for fibrous composites could be applicable to the 3D-printed PLA specimens. Static failure loads of 3D-printed PLA specimens with a hole and subjected to remotely applied loading were predicted successfully using the failure criterion based on stress and stress gradient conditions [16].

Cyclic tests were conducted to examine the fatigue life of PLA specimens [22,23,24,25,26,27,28,29,30,31,32]. The majority of the studies investigated unnotched specimens [22,23,24,25,26,27,28] to plot their S-N curves while some of them examined the effect of the notch with cyclic loading including the effort to enhance the material properties of 3D-printed specimens [29,30,31,32,33]. However, to the best knowledge of the authors, there was almost no study on the tensile and cyclic failure of perforated PLA specimens by an inserted pin loading. Pin loading results in very different failure loads from remotely applied loading.

The objective of this study was to understand and predict the failure of an orthotropic material like 3D-printed PLA specimens subjected to either tensile or cyclic loading using an inserted pin. The focus of the study was to determine the failure loads and predict those rather than to further investigate the progressive failure mode and mechanism. The print angles of the PLA specimens were varied to examine the effect of different failure modes on both tensile and cyclic failure. The recently proposed failure criterion was used to predict the failure load and path of the orthotropic PLA specimens under pin loading.

The next two sections describe the preparation of the 3D-printed PLA specimens and the test procedures. Then, the finite element analysis and the failure criterion are presented to predict the failures, which is followed by the experimental results of both tensile and cyclic loading, a discussion, and a prediction of the experimental results. At the end, the summary and conclusion are provided.

## 2. Preparation of Specimens

All specimens were generated using a 3D printer. The S5 printer from UltiMaker© (Utrecht, Netherlands) was used for this purpose. The material used was PLA Silver Metallic, also from UltiMaker©. Although the printer can use two nozzles, only one AA 0.4 nozzle was used. The specific printing parameters used for all specimens are shown in Table 1. The infill flow of 110% was selected here, which meant that slightly more material was extruded than originally calculated by the slicer software. Since only infill is printed for the specimens and no wall areas are used, problems may arise when calculating the required print lines within a layer. A slightly increased material flow was selected to compensate for the resulting under-extrusion.

Different print angles were used throughout the experiments as listed in Table 2. Symmetric print angles with respect to their midplanes were used for all the specimens. There were multiple test series with varying print angles. The print angle was measured in terms of the direction of the load. Thus, the print angle of 0° means the print angle is aligned with the direction of the applied load. Print angles varied from 0° to 90° for different specimens. The specimen ±θ° means each print layer was altered from +θ° to −θ° and vice versa, while all the layers are symmetric about its midplane.

**Figure 1:**
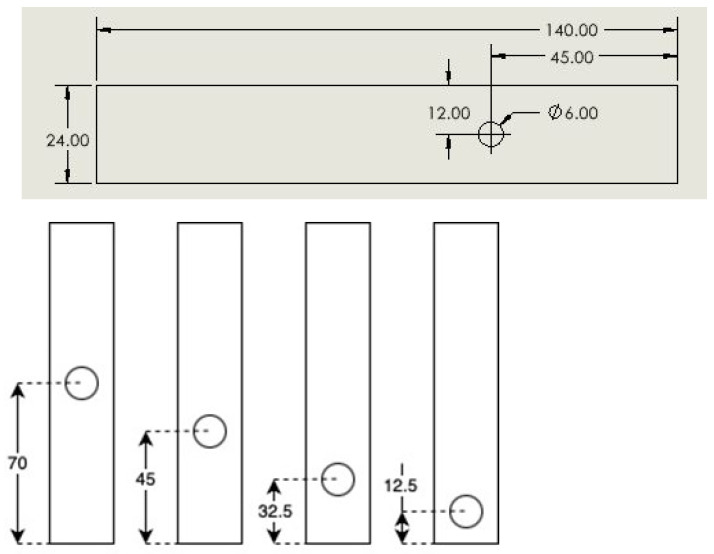
(**Top**) Specimen geometry, and (**Bottom**) four different hole locations in millimeters.

The standard printed samples had a length of 140 mm, a width of 20 mm, and a nominal thickness of 2 mm unless stated otherwise. The ASTM standards were not used for the present specimen’s geometry. After the specimens were printed, a hole was drilled into the specimens at four different locations, with a nominal diameter of 6 mm. However, since the hole needs a slight clearance for the pin of 6 mm diameter, the actual hole diameter was set to 6.35 mm. Figure 1 shows the specimen geometry and four different locations of the holes. The reason for different hole locations was to examine the effect of the distance from the hole to the edge of the specimen on potentially different failure modes such as net-tension, shear-out, and bearing failure under inserted pin loading. In general, as the hole is closer to the edge of the specimen and the print angle is closer to the 0°, the possibility for shear-out or bearing failure modes increases. The specimens have a grip area of 20 mm long from the left end of the top figure or the top end of the bottom figure of Figure 1, while the other side is loaded by an inserted pin for the tensile loading.

## 3. Experimental Procedures

The MTS© 858 tabletop system was used to undertake all tests. Tensile tests were carried out at least three times for each geometry to determine the average failure stress. If the first three test data are consistent with a small standard deviation, no additional test was conducted for the same case. Otherwise, the test was repeated until there was a representative average value. The majority of the test data were very consistent with a small standard deviation for the first three specimens. The failure stress was computed from the maximum applied load at failure divided by the cross-section at the gripping site, i.e., without considering the hole. A 2 mm/min displacement rate was selected for the static tensile tests. For cyclic pin loading, the settings used are shown in Table 3.

Percentages of failure stress were used as the maximum load in the cyclic tests. The other values, such as average load, amplitude, and minimum load, were determined using the R-value which is the ratio of the maximum stress to the minimum stress of the cyclic loading. It should be noted, however, that the forces applied by the test equipment were slightly lower than the user-specified applied forces under the force-controlled cyclic testing with a relatively high loading rate. Thus, the applied stresses for the cyclic loading in the S-N data were from the measured applied forces.

**Figure 2:**
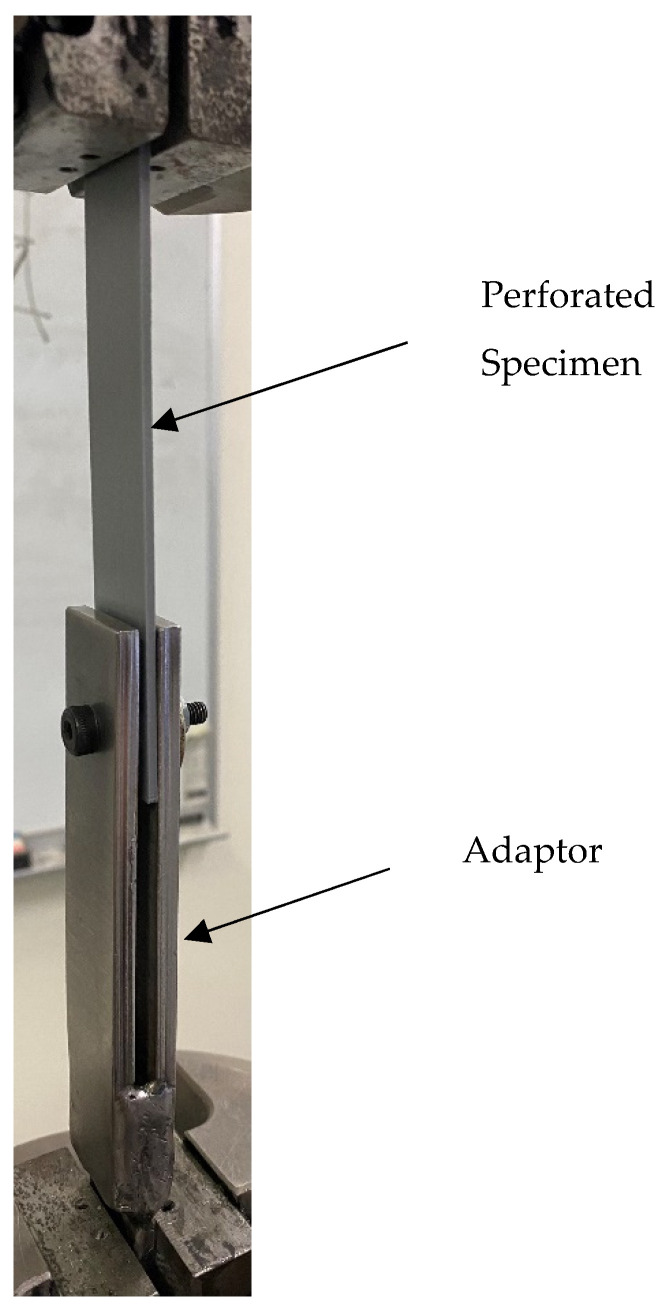
Specimen with inserted pin loading.

Figure 2 shows the adaptor at the lower side used to apply the pin loading to a specimen, which is already mounted in the test machine. The adaptor was made of steel, which is much stiffer than PLA. When inserting the specimens, it is important to always orient them such that the top side of the bottom figures of Figure 1 is gripped directly by the MTS machine, while the bottom side is loaded by an inserted pin. Attention should also be paid to the correct alignment of specimens with the pin loading mechanism and the correct tightening of the pin so that there is no major movement that could influence the measurement.

## 4. Finite Element Analysis and Failure Criteria

**Figure 3:**
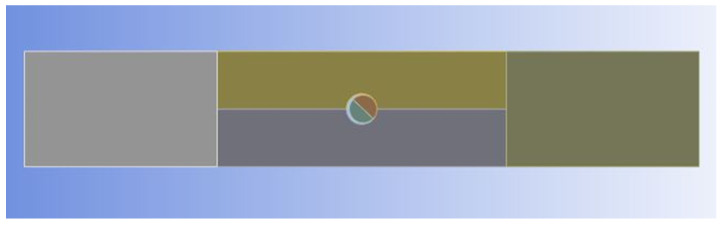
Computer-aided design model for a specimen with an inserted pin.

A Finite Element Analysis (FEA) program, Ansys©, was used to model the tested specimens. The geometries were created using SolidWorks© software (version 2022.2.0), a computer-aided design tool. Figure 3 shows the combined geometries of the perforated specimen and the pin. These were created as separate solid parts and combined using the SolidWorks assembly tool. Here, it was ensured that the pin was aligned so that there was at least one point of contact with the specimen hole for the tensile loading to avoid any rigid body motion in the static analysis. The contact boundary condition was applied between the pin’s boundary and the inner boundary of the hole. Different colors only indicate different sub-geometries of the overall component, and there is no further meaning.

The 3D-printed PLA specimens showed very different failure strengths along the printing direction and its transverse direction. However, their elastic moduli were not very different. As a result, the PLA specimens were assumed to be isotropic in terms of their stiffness for the FEA as the first order approximation. However, the anisotropic strengths were considered in the post-computation of the analysis when the failure criterion was applied. The hole location of 70 mm was simulated in all numerical analysis. The required material properties of the PLA were determined by four tensile tests of standard test coupons without holes and can be seen in Table 4. The test data was very consistent with those obtained in prior studies.

The tensile load was applied to the left boundary of Figure 3 by applying the uniform displacement in the direction of the specimen length to emulate the tested loading condition. The pin was withheld from any displacement. The pin was modeled as steel with given data from the Ansys workbench©. Frictional contact conditions with a frictional coefficient of 0.2 were selected for the contact surfaces between the pin and the hole surface in the specimen [34,35]. A previous study [17] also assumed the same frictional coefficient, which resulted in a very close contact surface between the experimentally measured and numerical predicted results.

**Figure 4:**
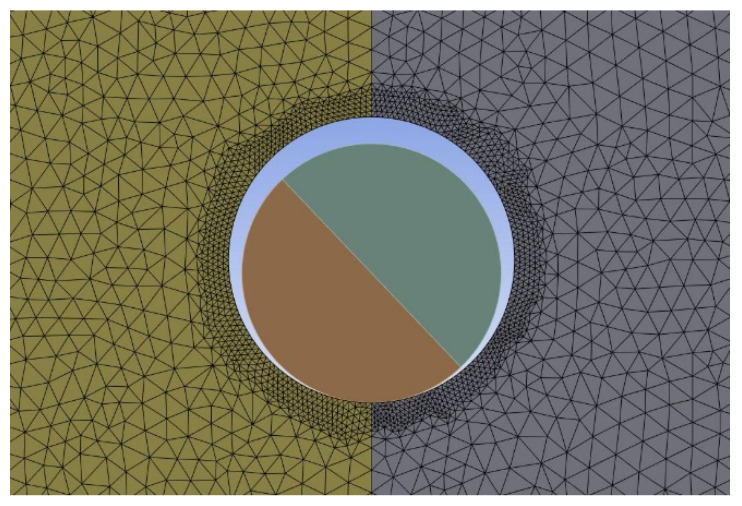
Finite element mesh of the pin and the specimen around the pin.

In the FEA, a mesh size of 0.5 mm was initially applied to both specimens and pins. Then, a mesh sensitivity study was conducted in terms of accuracy and computational efficiency. In the end, a refined mesh as shown in Figure 4 was used around the hole to maintain accuracy at the contact surface because the accurate contact stress is important to predict the failure of the specimens.

Numerical analyses were conducted for the print angles 0°, ±45°, and 90°. A hole location of 70 mm was used here in every case. This was to compare the experimental failure loads with the analytical predictions utilizing the stress- and stress gradient-based failure conditions presented in [14,15,16,17,18]. Once the stresses were obtained from the FEA, as modeled and described above, they were tested using the following failure criterion to determine the failure stress.

Because the failure stress is quite different between the 0° and 90° specimens, the failure criterion must be applied in two different directions: the print direction and its normal direction. To this end, a local coordinate system had to be established for each specimen. The 1-direction is along the print line, and the 2-direction is perpendicular to it and in the direction of the molten area between the individual print lines. This allowed stress to be defined for the two failure directions. For the failure between the print lines, the stress in the 2-direction $\sigma_{2}$ was selected as the effective stress $\sigma_{2eff}$. On the other hand, for the failure through the print lines, the effective stress was used as below:

(1) $$\sigma_{1eff}=\sqrt{\sigma_{1}^{2}+{\left(\frac{E}{G}\right)}^{2}\tau_{12}^{2}}$$

where $\sigma_{1}$ is the normal stress along the print direction and $\tau_{12}$ is the shear stress. In addition, *E* and *G* are the elastic and shear moduli, respectively.

The recently proposed failure criterion has two conditions to be met for failure. The first stress condition is shown in Equation (2). Here, the effective stress at one point must be greater than or equal to the failure stress. The effective stress $\sigma_{eff}$ must be selected from the simulation data either as $\sigma_{1eff}$ or $\sigma_{2eff}$, depending on the failure mode.

(2) $$\sigma_{eff}\geq \sigma_{fail}$$

where $\sigma_{fail}$ is the failure strength of each direction. The failure strength was determined from the standard tensile specimen without any holes.

The second condition for the stress gradient, which must be fulfilled at the same point simultaneously with the first condition for material failure to occur, is given in Equation (3).

(3) $$\sigma_{eff}\geq {\left(2EY\frac{d\sigma_{eff}}{ds}\right)}^{\frac{1}{3}}$$

Here, *E* is the elastic modulus, and *Y* is a material constant which is equivalent to the critical energy release rate of the material. The derivative of the stress over the failure path *s* is the stress gradient. To determine the material constant *Y*, experimental testing was conducted on any perforated specimens with the same printing directions using remotely applied tensile loading. Then, FEA was performed to determine the material constants for the stress gradient condition using Equation (3) for the printing directions investigated. Those material constants were used to predict failure subjected to inserted pin loading. Remotely applied loading and pin loading result in very different stress distribution, stress concentrations, and failure locations, even for the same specimens, which lead to completely different failure loads and failure paths.

## 5. Experimental Results

### 5.1. Tensile Tests

**Figure 5:**
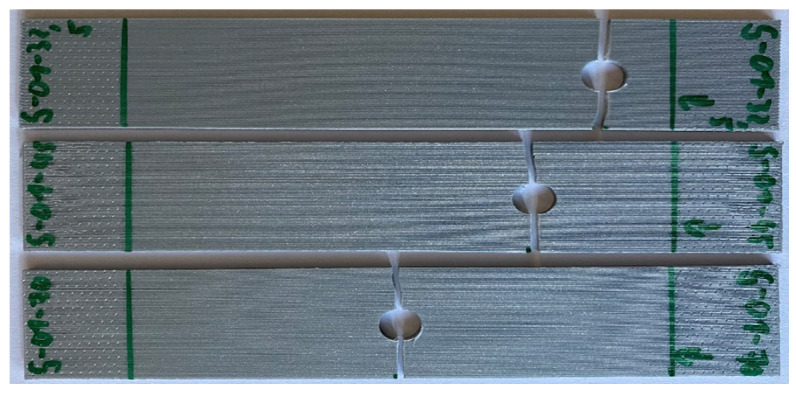
Failure of 0° specimens with different hole locations under remote loading.

**Figure 6:**
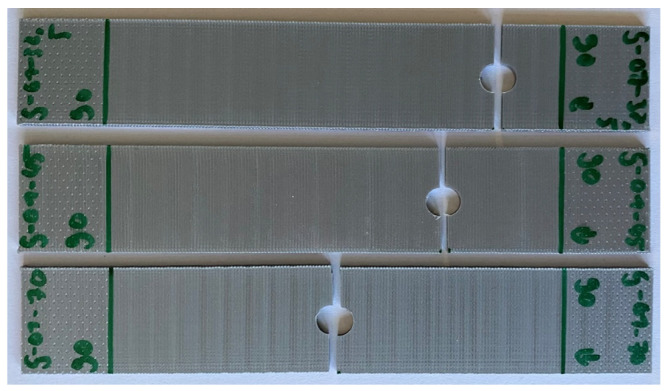
Failure of 90° specimens with different hole locations under remote loading.

First, the failure stress was compared between the inserted pin loading and the remote loading, which did not use the pin. In the latter case, tensile loading was applied to the perforated specimens directly at both ends of the specimens. Figure 5 and Figure 6 compare the failure modes of the perforated 0° and 90° specimens with different hole locations subjected to remote loading. Specimens under remote tensile loading failed consistently at the midsection of the hole in the perpendicular direction to the loading regardless of the hole locations or the printing angles. All the figures, unless mentioned otherwise, were obtained from the most representative specimens of at least three repeated test results.

**Figure 7:**
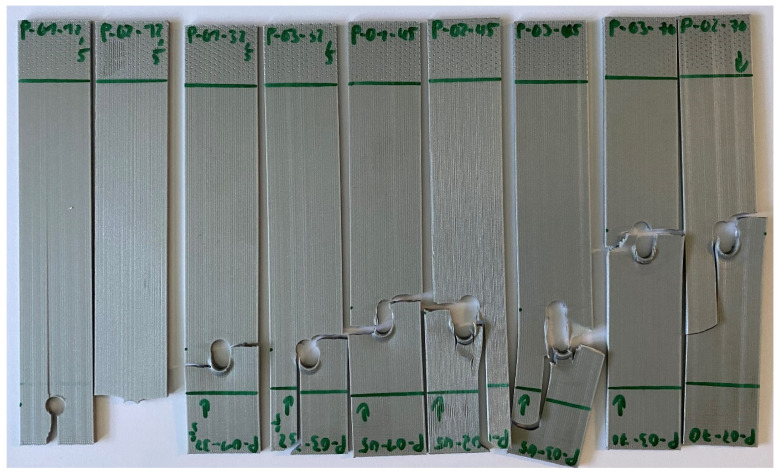
Failure of 0° specimens with different hole locations under pin loading.

**Figure 8:**
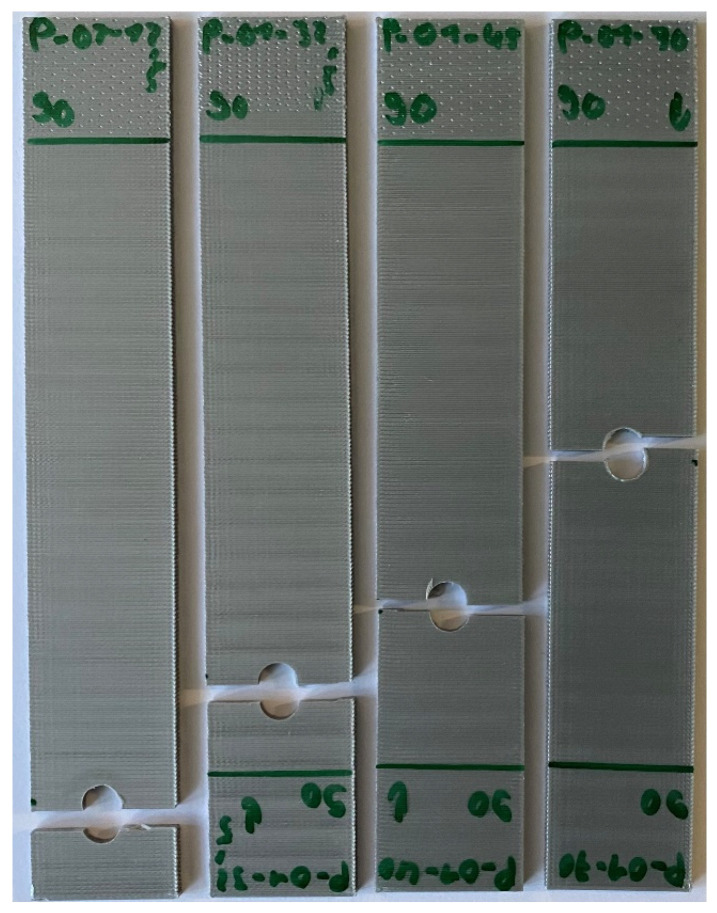
Failure of 90° specimens with different hole locations under pin loading.

Figure 7 and Figure 8 show the failed specimens with inserted pin loading. When the print angle is 90°, failure occurs between the print lines, so the failure direction is perpendicular to the loading direction like the remote loading case. However, the 0° specimens resulted in very different failure modes depending on the hole locations. All failure modes like net tension, shear-out, and bearing failures are shown in Figure 7. Bearing failure occurred for the hole at the location of 12.5 mm in Figure 1.

**Figure 9:**
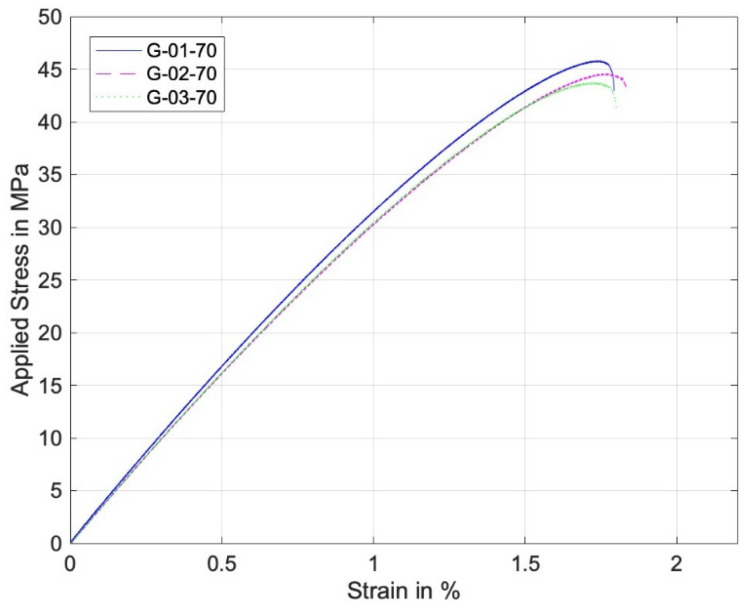
Stress–strain curve of perforated 0° specimens under remote tensile loading.

**Figure 10:**
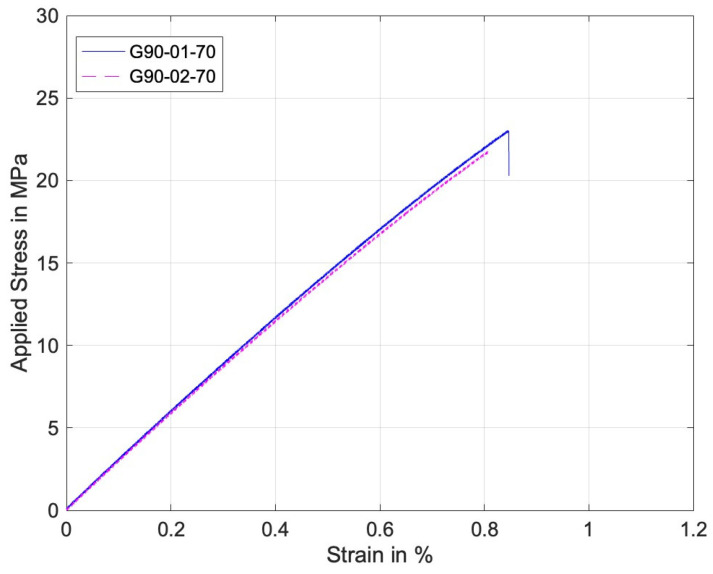
Stress–strain curve of perforated 90° specimens under remote tensile loading.

Figure 9 and Figure 10 are the stress–strain curves of the perforated specimens of 0° and 90° print angles under remote tensile loading. The stress and strain were computed without considering the hole, per the standard tensile coupon. Both curves are quite linear up to the final failure. The failure stress of the 0° specimen was much higher than that of the 90° specimen.

**Figure 11:**
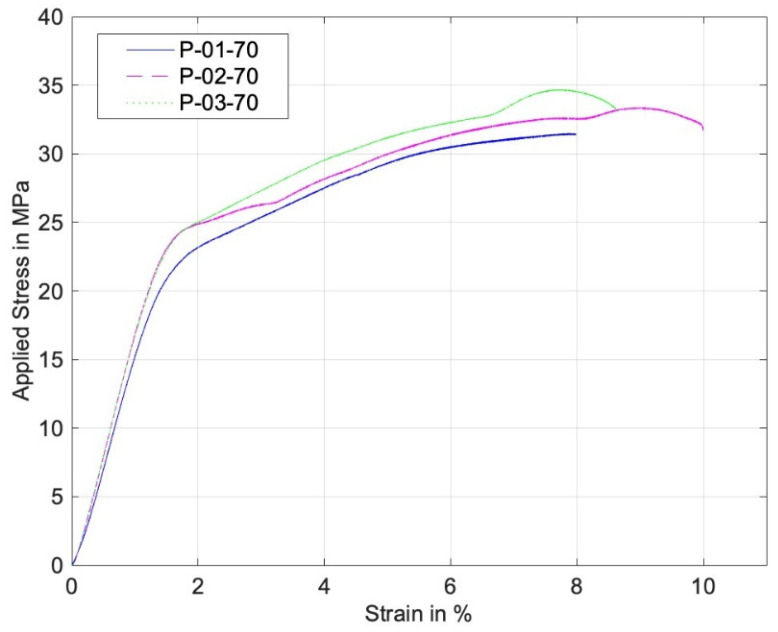
Stress–strain curve of perforated 0° specimens under pin loading.

**Figure 12:**
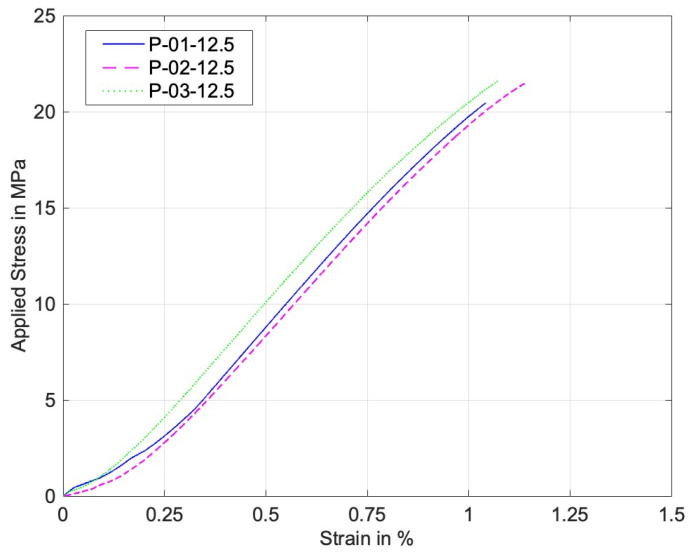
Stress–strain curve of perforated 90° specimens under pin loading.

**Figure 13:**
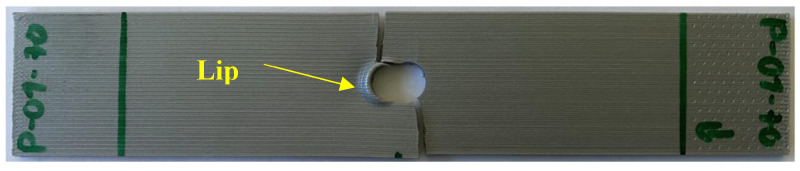
Lip formed during the contact loading between the pin and perforated PLA specimen.

On the other hand, Figure 11 and Figure 12 are similar stress–strain curves under pin loading. The 0° specimens under pin loading show bilinear curves. The major reason for the second linear part of the graphs was the formation of lips at the hole during the contact loading by the harder pin, which resulted in a localized deformation until the final failure. Figure 13 shows a lip formed due to the contact between the pin and the perforated PLA specimen. Thus, the strain during the formation of the lip increased with a small increase in the stress. The 90° stress–strain curves show a minor knee at the early stage. This resulted from the contact between the pin and the specimens. The first part of the graphs is before the formation of the complete contact while the second part is after the complete contact. Regardless of the knee, the overall stress–strain curves were quite linear.

**Figure 14:**
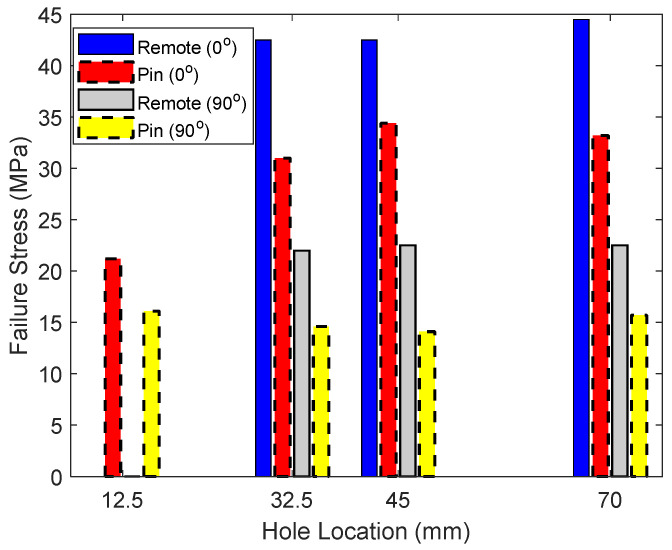
Comparison of failure stresses of 0° and 90° specimens under remote and pin loading.

The failure stresses of 0° and 90° specimens were compared in Figure 14 for four different hole locations. Both remote and pin loadings were included in the figure. When the hole was located at 12.5 mm from one end of the specimen, remote loading could not be applied because of the unavailable grip section. Thus, the data is not available in the figure. The failure stress under remote loading was always greater than that under pin loading because pin loading results in a much higher stress concentration than the other. The strength was not much different from one another for different hole locations, except for the case of the hole location 12.5 mm of the 0° specimen under pin loading. The major reason for this is the shift of the failure mode to bearing failure for the 12.5 mm-holed specimens.

Finally, Table 5 is the summary of all failure stresses of specimens under pin loading. All print angles were listed in the table. The hole location was varied for the 0°, 45°, and 90° specimens. However, the hole was always in the middle of all other specimens. As noted in the table, the failure stress decreased along with the increase in the print angle because the print lines could carry the largest load. The specimens of ±15° print angles were exceptional. Their failure stress was slightly lower than that of ±30° print angles. This was against the theoretical prediction. It is not clear at this point what the cause was. However, because the deviation was not significant, any further investigation was not conducted to focus on other aspects of the research.

### 5.2. Cyclic Tests

**Figure 15:**
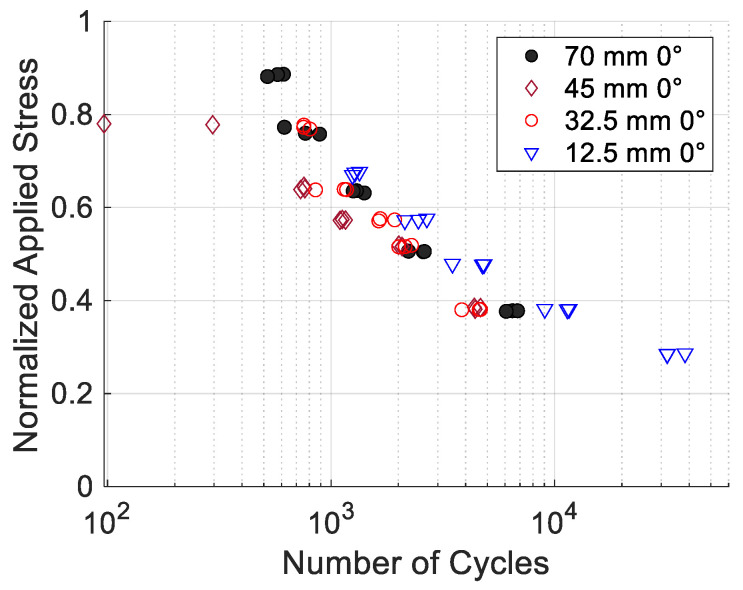
Fatigue life curves for 0° specimens under pin loading.

**Figure 16:**
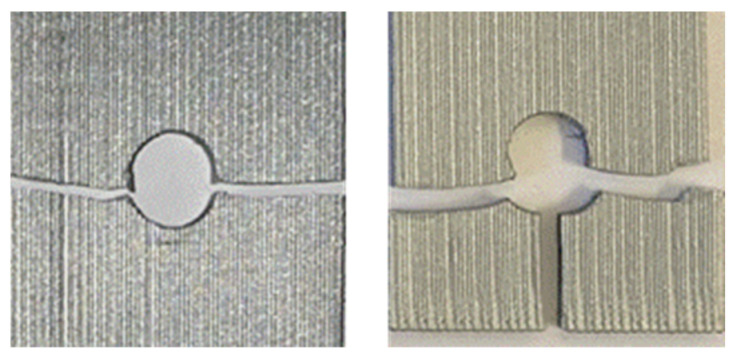
Failure mode for 0° printing orientation (**left**: 70, 45, 32.5 mm hole location; **right**: 12.5 mm hole location).

**Figure 17:**
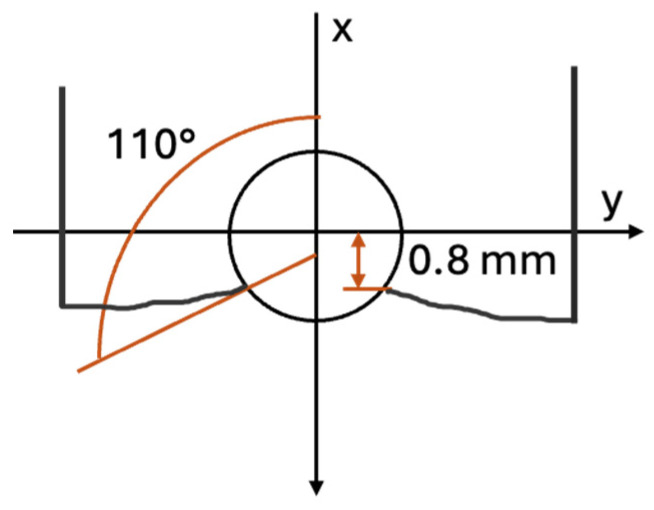
Measurement for failure initiation location and orientation.

All the cyclic tests were conducted using inserted pin loading as tension–tension. The testing had the load ratio *R* = 0.1, i.e., the maximum load is ten times greater than the minimum load. The fatigue life, i.e., the number of cycles to failure, was plotted in Figure 15 for the 0° specimens with four different hole locations. This is called the S-N data or curve hereafter. The applied stresses for the cyclic loading were normalized with respect to their respective static failure stresses of the same perforated specimens. Figure 15 shows that the data points for different hole locations are clustered. In particular, the holes at 70 mm and 32.5 mm show very close data. The data for the 45 mm hole shows a notable deviation from the normalized stress level of around 0.8. The reason for this is not clear, and further study is needed. The hole at 12.5 mm generally indicates a slightly larger number of cycles to failure than other holes under the same normalized stress level. This seems to be related to a different failure mode of the hole at 12.5 mm. Figure 16 compares the failure modes of 0° specimens of different hole locations and subjected to cyclic pin loading. The hole at 12.5 mm had a different failure mode than other hole cases. To trace the failure more closely, the location of the failure initiation and its orientation were measured, as shown in Figure 17. The vertical distance was measured from the midline of the hole, and the angle was measured with respect to the applied loading direction.

**Figure 18:**
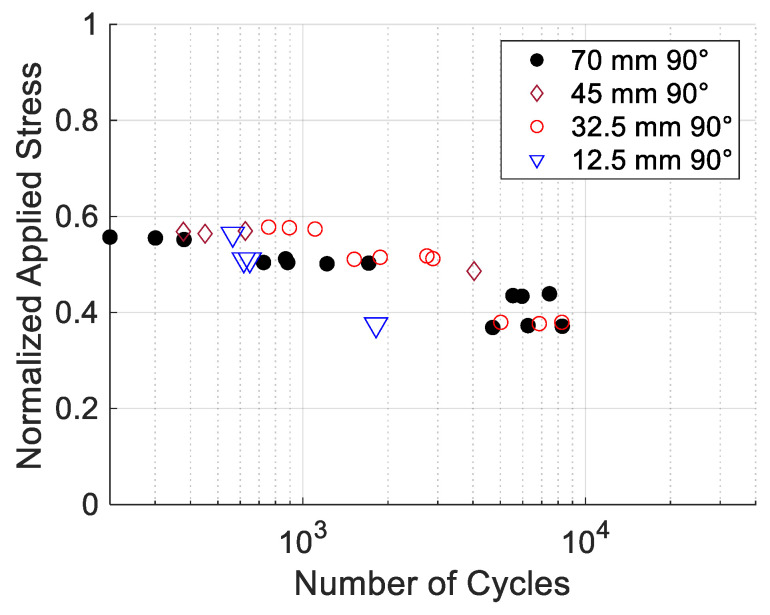
S-N data for 90° specimens under pin loading.

The cyclic test results for the 90° specimens with different hole locations are plotted in Figure 18. It is worth noting a couple of points. If the normalized applied stress level was higher than 0.6, the number of cycles to failure was very small. As the stress level decreased to less than 0.6, the number of cycles to failure decreased very gradually. All the data were quite clustered except for one data point for the hole at 12.5 mm.

Comparing 0° to 90° specimens with different hole locations, the hole locations did not make a large difference in the S-N curves. However, the print angles resulted in quite different S-N curves, even in terms of the normalized applied stress. That is, the print angle plays an important role in cyclic failure under pin loading. Thus, the next study was to compare the S-N curves for different print angles. All the cases had the hole location at 70 mm.

**Figure 19:**
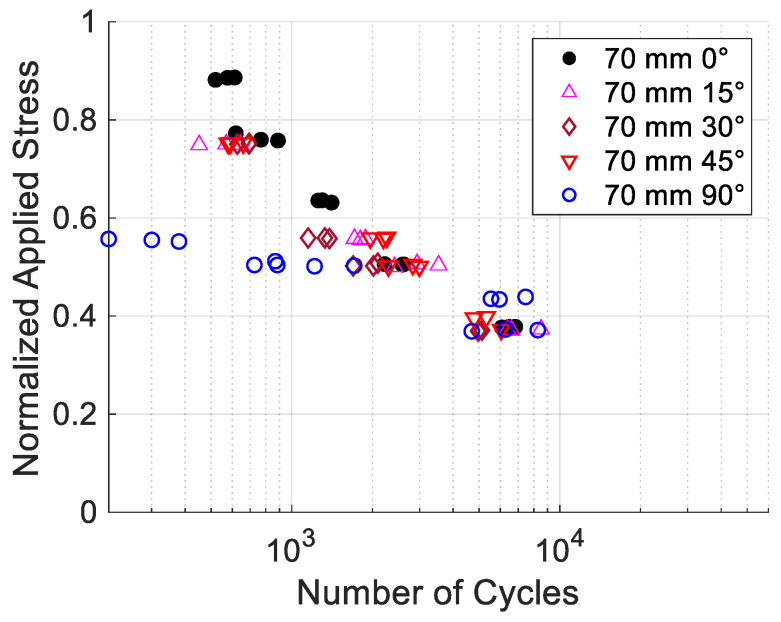
S-N data for printing angles for ±15° to ±45°.

**Figure 20:**
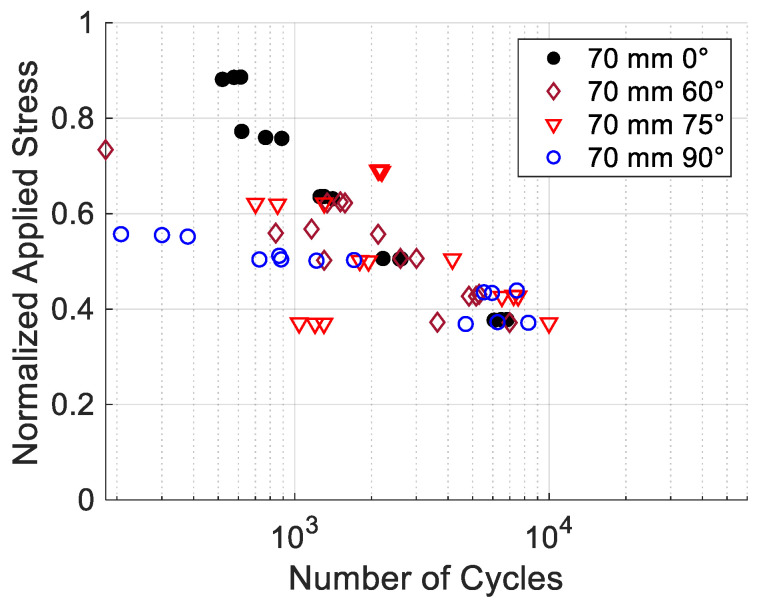
S-N data for printing angles for ±60° and ±75°.

**Figure 21:**
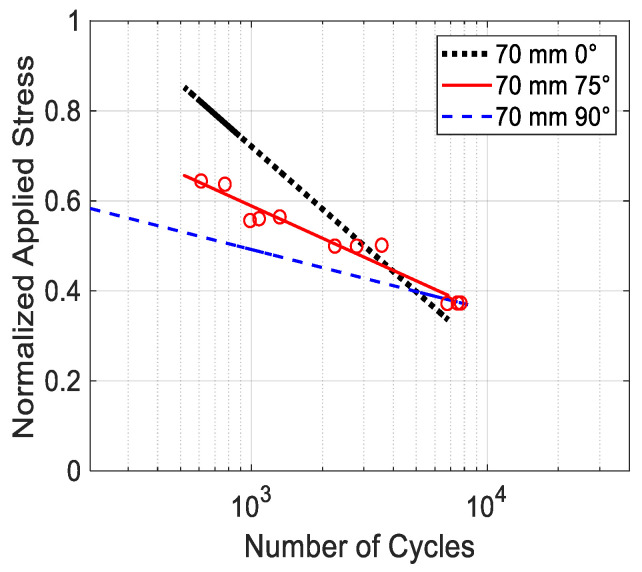
S-N data for printing angle 75°.

Figure 19 compares the S-N data for the printing angles of ±15°, ±30°, and ±45°, while Figure 20 shows the data for ±60° and ±75°. All the figures include the data for 0° and 90° as the upper and lower boundaries. The S-N data in Figure 19 show that the results are very clustered among ±15°, ±30°, and ±45°, and they are not much different from the data for 0°. On the other hand, Figure 20 shows the S-N data are more scattered for the ±60° and ±75° specimens. In particular, the ±75° specimens had very scattered data. To understand this, specimens with only a +75° print angle were printed without a −75° print angle. These specimens are not symmetrical about the loading axis. The cyclic test data were plotted in Figure 21 along with their best-fitted line. For comparison, the best-fitted lines for the 0° and 90° specimens were also plotted together in the same figure. When the specimen had only a +75° print angle without −75°, the test data were quite consistent, and without much scattering, and the best-fitted line was well bounded between the lines of the 0° and 90° specimens. The failure was between the print lines. However, when the specimens had both +75° and −75° print angles, the failure path had to cross through one of the print angles. That process seemed to result in very scattered cyclic test results.

**Figure 22:**
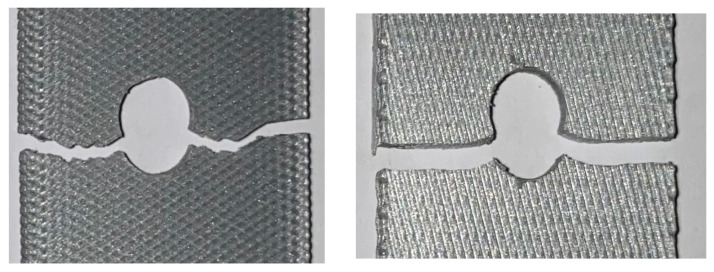
Failed ±15° (**left**) and ±30° (**right**) specimens after cyclic loading.

The failed specimens after cyclic loading are shown for the ±15° and ±30° specimens in Figure 22. Table 6 summarizes the initial failure locations and orientations for all the specimens with holes in terms of the measurement shown in Figure 17. Among all the specimens, the 90° specimens had more consistent failure locations and angles. The failure was initiated at the vertical distance between 0.7 mm and 0.8 mm with the 90° failure orientation. This results from a failure between the print lines. Other specimens had wider variations in the initial failure locations and failure orientation because they were involved with the through-the-print-line failure.

**Figure 23:**
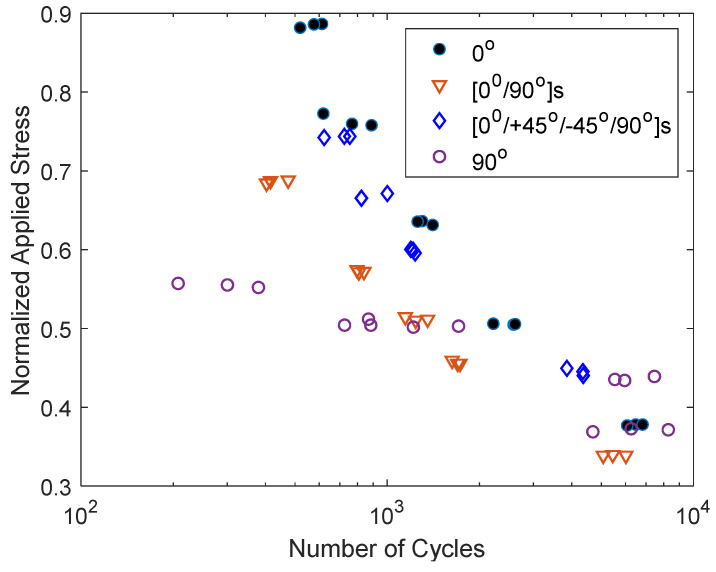
S-N data for [0°/90°]_s_ and [0°/±45°/90°]_s_ specimens.

The results for the combined print angles are presented next. First, the results for the [0°/90°]_s_ and [0°/±45°/90°]_s_ specimens are shown in Figure 23. To compare the results, both 0° and 90° specimens were also included in the figure. The plot shows that the [0°/±45°/90°]_s_ specimens had a greater number of cycles to failure for a given normalized applied stress than the [0°/90°]_s_ specimens. Furthermore, the former had S-N data close to the ±45° specimens, which was much closer to the 0° specimen data than the 90° specimen data. On the other hand, the [0°/90°]_s_ specimens had cyclic test data in the middle of the 0° and 90° test data. The [0°/90°]_s_ and [0°/±45°/90°]_s_ specimens showed a clean failure section normal to the loading axis like the 90° specimens.

## 6. Prediction

The static failure stresses of the perforated PLA specimens subjected to pin loading were predicted from the FEA results using the failure criterion discussed previously. To this end, both effective stresses $\sigma_{1eff}$ and $\sigma_{2eff}$ were computed, as well as their gradients depending on the failure mode. Table 7 shows the theoretically predicted failure stress of perforated PLA specimens subjected to pin loading. The 0°, ±45°, and 90° specimens were compared in the table. The effective stress $\sigma_{1eff}$ was used for the 0° and 45° specimens, while $\sigma_{2eff}$ was used for the 90° specimens because of their different failure modes. The failure stress was determined by the applied load at failure divided by the cross-sectional area across the whole specimen width without holes at the gripping site of the testing machine. The predicted failure stresses agreed very well with the experimental data for the pin loading. Furthermore, the location and orientation of the failure initiation are also given in Table 7, and their predicted results are also very comparable to the experimentally observed results.

The failure strength of different printed angles was also predicted using the following equation:

(4) $$\sigma_{\theta }=\sigma_{90}+\left(\sigma_{0}-\sigma_{90}\right)\cos\theta$$

where $\sigma_{0}$ and $\sigma_{90}$ are the strength of the 0° and 90° specimens, and $\sigma_{\theta }$ is the failure strength of the ±θ° specimens. Table 8 compares the predicted failure stress by Equation (4) to the experimental results. When predicting $\sigma_{\theta }$, the predicted $\sigma_{0}$ and $\sigma_{90}$ were used. Equation (4) yields very reliable failure strength of specimens with any printing angles of ±θ°. The largest error was for ±15° specimens, and it was 9.2%. As stated in the previous section, the data set for the ±15° specimens are considered outliers. All other print angles had an error of less than 5% consistently.

Equation (4) cannot be used directly for specimens with multiple different print angles like [0°/90°]s and [0°/±45°/90°]s. However, Equation (4) can be used for each print angle of the specimens and their mean value can be determined. The predicted mean failure stress of [0°/±45°/90°]s specimens was 25.5 MPa, which is very close to their experimental failure stress of 25.6 MPa. However, the predicted failure stress of [0°/90°]s specimens was 23.8 MPa, while the experimental failure stress was 27.7 MPa. Therefore, Equation (4) did not accurately predict the failure stress of [0°/90°]s specimens. Instead, the failure stress of [0°/90°]s specimens was close to that of the ±45° specimens. One possible reason is that, when a 0° layer was printed on top of an existing 90° layer, the weak interface of the print lines of the prior 90° layer was strengthened during the 0° print.

**Figure 24:**
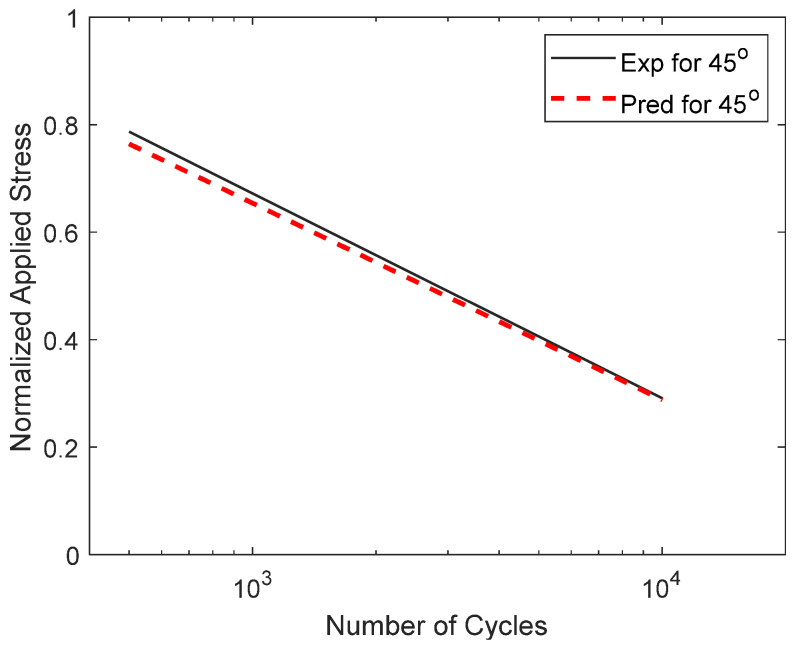
Comparison of S-N curves between experimental and predicted results for ±45° specimens.

The S-N curves of the perforated PLA specimens subjected to cyclic pin loading were also predicted using the failure criterion and Equation (4). The two different failure modes, through and between print lines, also affected the S-N curves of the perforated PLA specimens. They had very distinguishable S-N curves even though the applied stress was normalized to its failure stress. This is because the cyclic fatigue behavior differs between the two failure modes. Thus, the S-N curves for the 0° and 90° must be determined from a series of cyclic tests. Then, the S-N curve for an intermediate angle ±θ° was predicted using Equation (4). To do that, the best linear fit was conducted for the cyclic test data of the 0° and 90° specimens, respectively. Then, Equation (4) is applied to the equations for the curves between the two angles. Figure 24 compares the S-N curves between the experimental and predicted results for the ±45° specimens. The experimental curve is the best linear fit of the experimental data of the ±45° specimens, while the predicted curve was obtained with Equation (4) using the 0° and 90° S-N curves. The predicted curve compares well with the experimental curve. Similar comparisons were made for other angles, and they agreed well overall, even though the accuracy was better for one angle than for another angle.

**Figure 25:**
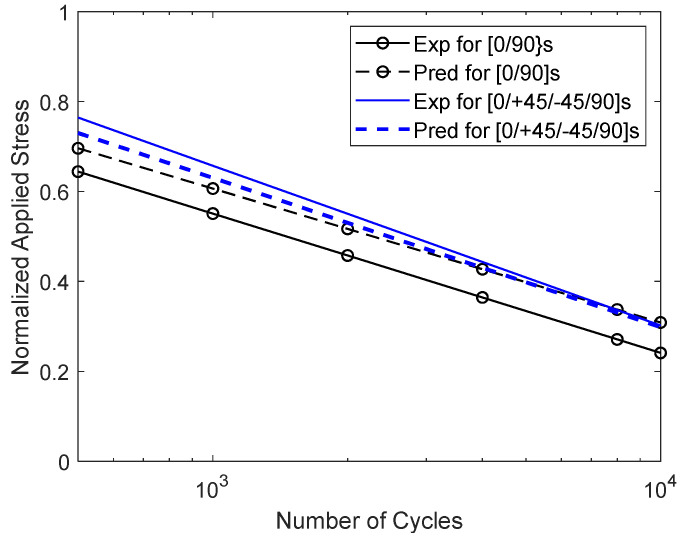
Comparison of S-N curves between experimental and predicted results for [0°/90°]s and [0°/±45°/90°]s specimens.

The S-N curves were also predicted as the static failure stresses for the [0°/90°]s and [0°/±45°/90°]s cases using Equation (4), as shown in Figure 25. Like the static failure stress, the predicted S-N curve was close to the experimental curve fit for the [0°/±45°/90°]s specimens. On the other hand, there was a larger discrepancy between the prediction and the experimental results for the [0°/90°]s specimens. This could be explained in a similar way, as stated for the static failure stresses. Even though the failure stress of the [0°/90°]s specimens was close to that of the ±45° specimens, the S-N curve of the former was not close to that of the latter. Instead, the S-N curve of the [0°/±45°/90°]s specimens was close to that of the ±45° specimens.

## 7. Summary and Conclusions

Perforated 3D-printed PLA specimens were investigated using inserted pin loading for both tensile and cyclic fatigue failure. The specimens had various print angles like ±θ°, [0°/90°]s, and [0°/±45°/90°]s. The hole location was also varied along the length of the specimens. Some had holes at the center of the specimens and others had holes closer to one end of the specimens.

The experimentally measured tensile failure stresses of the perforated specimens were compared between pin loading and remotely applied loading. As expected, the pin loading resulted in much lower failure stresses than the remotely applied loading because of higher stress concentration at the holes resulting from the former. In addition, the pin loading initiated the failure away from the location of the minimum cross-section of the specimen, which is the midsection of the holes perpendicular to the loading because of the shift in the major stress concentration location.

The recently proposed failure criterion, which uses both stress and stress gradient conditions to determine the failure, predicted the failure stresses under pin loading very reliably. Furthermore, the failure criterion also predicted well the locations and orientations of the failure initiation under pin loading as compared to the experimental measurements. The failure stress of the perforated 0° specimens was more than twice as large as that of the perforated 90° specimens. Hence, a change in print angles resulted in different failure stresses. The failure stresses of specimens with different print angles could be predicted using either the failure criterion or Equation (4) if the failure stresses were already determined for the 0° and 90° specimens. The failure stresses of the [0°/90°]s and [0°/±45°/90°]s specimens were computed by taking the average of the predicted failure stress of each printed layer. The latter specimens resulted in a very accurate failure stress as compared to the test results. However, the former specimens did not predict the failure stress comparable to the experimental data. Instead, the failure stress of the [0°/90°]s specimens was very close to that of the ±45° specimens.

There are two different failure modes: failure through or between the print lines, respectively. The 90° specimens had a complete failure between the print lines and showed a very clean failure section normal to the loading direction. All other specimens went through failure across the print lines at least partially or completely depending on the print angles and layers. If the specimens had print angles other than 0° and 90°, their failed sections showed some zig-zag shapes except for the [0°/±45°/90°]s specimens. In this case, the 0° and 90° layers seemed to dictate the overall failure process even though they contained ±45°.

Like the tensile failure stress, the S-N data for the 0° and 90° specimens under pin loading were very different, even though the cyclically applied stresses were normalized with respect to their tensile failure stresses. This suggests that not only the tensile failure stress but also the damage accumulation through cyclic loading are very different between the 0° and 90° specimens. The 90° specimens showed much lower normalized fatigue failure stress than the 0° specimens until approximately 7000 cycles. However, because the S-N data showed a much more negatively steeper curve for the 0° specimens, the normalized fatigue failure stress became larger for the same number of cycles for the 90° specimens after around 7000 cycles.

The S-N data of the specimens with the print angles ±15°, ±30°, and ±45°, respectively, were relatively close to the data of the 0° specimens, as the applied stresses were normalized to their respective static failure stresses. All that data had very small scattering. Thus, a single S-N curve may be used for those print angles with a reasonably permissible range of errors. The S-N data similarity results from the same dominant failure mode, failure through print lines.

However, as the print angles were ±60° and ±75°, the normalized S-N data were very different from the previous S-N data. The data were very scattered and mostly around the middle of those of the 0° and 90° specimens. Those specimens were influenced by both failures through and between print lines. On the other hand, the specimens with print angles of +75° but not −75° showed much fewer scattered data than those of ±75°, and their best-fitted S-N line was approximately in the middle of the 0° and 90° S-N curves. This is because the asymmetrically printed specimens had only failure between the print lines, like the 90° specimens.

Once the S-N curves for the 0° and 90° specimens were obtained from experiments, S-N curves of specimens with other print angles were also predicted using Equation (4), which was used to predict the static failure stresses. The predicted S-N curves agreed well with the experimental curves. Thus, Equation (4) was useful in predicting both static failure stresses and the S-N curves of specimens with various print angles.

The study showed that two different failure modes in 3D-printed PLA specimens significantly influenced their static and cyclic failure stresses. The failure criterion based on both stress and stress gradient conditions could accurately predict the static failure stresses, as well as failure paths, of perforated PLA specimens with inserted pin loading. Equation (4) was found useful to predict both static and cyclic failure stresses of PLA specimens with various print angles.

The qualitative findings of this study could be appliable to other 3D-printed polymer materials which have similar orthotropic failure strengths. It is also expected that the qualitative behavior would be similar for different hole geometries as long as the cyclic applied stress is normalized to its static strength of the given hole geometry because the failure criterion based on stress and stress gradient could reliably predict the failure strength of different hole geometries.

## Figures and Tables

**Table 1:** Printing parameters.

Print temperature	200 °C
Bed temperature	55 °C
Print speed	45 mm/s
Layer height	0.2 mm
Line width	0.35 mm
Infill density	100%
Infill flow	110%

**Table 2:** Overview of the test series.

Print Angle (°)	Hole Location (mm)
0	70/45/32.5/12.5
±15	70
±30	70
±45	70/32.5
±60	70
±75	70
90	70/45/32.5/12.5

**Table 3:** Cyclic test setup.

Loading frequency	0.5 Hz
Load ratio, *R*	0.1
Load variation	Sinusoidal
Applied loads in terms of percent (%) of failure load	98, 81, 67, 60, 54, 46, 40%

**Table 4:** PLA properties.

Elastic modulus	2.919 GPa
Poisson’s ratio	0.375
Failure strength	44.0 MPa

**Table 5:** Failure stresses of perforated PLA specimens under pin loading.

Print Angle (°)	Hole Location (mm)	Failure Stress (MPa)
0	70	33.2
0	45	34.4
0	32.5	31.0
0	12.5	21.2
±15	70	29.7
±30	70	31.9
±45	70	27.5
±45	32.5	28.2
±60	70	25.8
±75	70	20.1
90	70	15.7
90	45	14.1
90	32.5	14.6
90	12.5	16.1
[0°/90°]_s_	70	27.7
[0°/±45°/90°]_s_	70	25.6

**Table 6:** Failure locations and failure angle for specimens with holes at 70 mm.

Print Angle (°)	Vertical Location (mm)	Failure Initiation Angle (°)
0	0.78–1.22	70–90
±15	0.50–1.20	80–100
±30	0.73–1.18	85–90
±45	0.73–1.02	50–60
±60	0.33–1.23	60–65
±75	0.63–1.13	75–90
90	0.70–0.90	90

**Table 7:** Predicted failure of specimens with holes at 70 mm by pin loading.

Print Angle (°)	Exp. Fail Stress (MPa)	Theor. Fail Stress (MPa)	Error (%)	Exp. Fail Location (mm)	Exp. Fail Angle (°)
0	33.2	32.1	−3.3	0.7	70
±45	27.5	28.5	3.6	1.2	70
90	15.7	15.4	−1.9	0.7	90

**Table 8:** Comparison between measured and predicted failure stress for pin loading.

Print Angle (°)	Measured (MPa)	Predicted (MPa)	Error (%)
±15	29.7	32.7	9.2
±30	31.9	30.9	3.2
±45	27.5	28.1	2.1
±60	25.8	24.6	4.9
±75	20.1	20.2	0.5

## Data Availability

All the data were provided in this paper in the form of figures and tables.
